# Recovery of Renal Function in Clinical Patients with Acute Kidney Injury: Impact on Mortality

**DOI:** 10.3390/life12060852

**Published:** 2022-06-08

**Authors:** Tayse Tâmara Paixão Duarte, Marcia Cristina Silva Magro

**Affiliations:** Nursing Department, College of Ceilândia, University of Brasília, Brasília 70910-900, Brazil; marciamagro@unb.br

**Keywords:** Acute Kidney Injury, mortality, renal recovery

## Abstract

Objective: To assess the different renal function recovery patterns and their impact on the mortality of non-critical patients with hospital-acquired Acute Kidney Injury. Design: A prospective cohort study was conducted from January 2017 to December 2019. Methods: The patients included were those with Acute Kidney Injury acquired during their hospitalization, identified from Kidney Disease: Improving Global Outcomes (KDIGO). Renal function recovery was calculated through the serum creatinine ratio in relation to baseline creatinine at the renal function evaluation moment. A descriptive analysis of the results was performed, and the Backward method was adopted for the multivariate analysis. Results: One-thousand five-hundred and forty-six patients were evaluated in the medical clinic and 202 (13.06%) were identified to have Acute Kidney Injury; among them, renal function recovery varied over the six months of follow-up with greater expressiveness in the second and third months (from 61.02% to 62.79%). Recovery was a protective factor against in-hospital death in the first (OR 0.24; 95% CI 0.09–0.61; *p*-value = 0.038) and sixth month of follow-up (OR 0.24; 95% CI 0.09–0.61; *p*-value = 0.003). Conclusions: The incidence of renal function recovery varied throughout the six months of follow-up and reached progressively high levels from the second to the third months. Renal recovery was a protective factor against mortality during the follow-up period.

## 1. Introduction

Acute Kidney Injury (AKI) is one of the most worrying complications in hospitalized patients, even in non-critical care units [1], due to its potential predisposition to unfavorable outcomes, such as chronicity of renal damage, lack of recovery, and mortality [2].

In addition to affecting millions of patients worldwide, AKI is responsible for prolonging hospitalization times and, sometimes, for increasing the need for hospitalization, outcomes that exert an impact on the costs of health services. Early recognition of the risk of developing AKI, despite its minor severity, is relevant because even the mildest forms of AKI can trigger serious consequences [3].

Studies are still under development to understand the mechanisms of renal function recovery [4] and the impact of renal recovery on the mortality rates in the short- and long-term, which are motivating conditions of this study.

It is known that maximizing renal function recovery for 48 and 72 h of AKI detection should be the goal of any treatment strategy for this syndrome [5], although there is still no consensus on the definition of renal function recovery. In addition to highlighting different renal recovery patterns (early sustained reversal, late sustained reversal, relapsed AKI and no recovery of renal function) after AKI, a cohort study evidenced that patients without sustained renal recovery evolve with higher mortality rates during hospitalization [4].

In the intensive care setting, the mortality detected at one year was five times higher among patients with AKI without recovery, when compared to those with a history of rapidly reversed AKI [6] and, in non-critical care, mortality after 12 months affected 64.9% of the patients who presented with AKI [6]. A cohort study indicates that, of 534 non-critical patients with AKI, 45% presented full renal recovery and that, among them, 9% died still while hospitalized [1].

Lack of consensus on a consistent definition of renal recovery has favored worse prognoses [7,8,9], which results in a higher limitation to evaluate the extent of renal impairment [10]. However, it is noted that renal recovery seems to exert an important impact on prognosis [11].

There is some evidence of renal recovery and mortality in intensive care units [2,12], but in non-critical care, there are few studies that address this relationship; consequently, it is believed that a better understanding of this condition would allow for more consistent care and, therefore, for the optimization of effective, preventive and individualized strategies as a way to mitigate risk and improve the clinical outcomes of hospitalized patients with AKI.

Provision of care for patients with AKI or at risk of renal impairment should occur on a continuum from admission through the different hospitalization scenarios, considering the possibility of mitigating the cost of care and achieving better prognoses for renal patients [13]; therefore, this study is justified by the need to verify the relationship between renal function recovery and the clinical outcome of patients in non-critical care units.

The objective of this study was to evaluate the different renal function recovery patterns and their impact on the mortality of non-critical patients with hospital-acquired Acute Kidney Injury (HA-AKI) in the short- and long-term.

## 2. Materials and Methods

A quantitative prospective cohort study was carried out from January 2017 to December 2019, with patients hospitalized in the medical clinic of a public, general and large-size hospital located in *Distrito Federal*, Brazil.

A total of 1546 patients were evaluated and 202 patients with acute kidney injury were identified, this being the study sample. During follow-up (6 months), sample loss occurred due to death and failure to collect biomarkers.

The patients included were those admitted to the medical clinic ward with a 24-h period of stay and sustained change in serum creatinine ≥0.3 mg/dL in relation to the baseline for at least 48 h after admission (stage 1 of the Kidney Disease: Improving Global Outcomes [KDIGO] classification). The patients excluded were those aged ≤18 years old, with an estimated glomerular filtration rate ≤30 mL/min/1.73 m^2^, undergoing hemodialysis or peritoneal dialysis prior to HA-AKI, renal transplantation and under palliative care.

Sample calculation considered 80% power and was obtained by the formula below [14]: $N=2\frac{{\left[z_{\alpha }2{\left(\bar{p}\bar{q}\right)}^{\frac{1}{2}}+z_{\beta }{\left(p1q1+p2q2\right)}^{\frac{1}{2}}\right]}^{2}\left(1+\left(n-1\right)\rho \right)}{n{\left(p1-p2\right)}^{2}}$.

The sample consisted of 202 patients with hospital-acquired Acute Kidney Injury—HA-AKI, after hospitalization in the Medical Clinic Unit. By definition, HA-AKI occurs after 24 hospitalization hours [15] and, in this study, its identification was based on the creatinine criterion from the KDIGO classification [16]. The urinary volume criterion was not used due to the inaccuracy and scarcity of urinary volume records of the patients hospitalized in the Medical Clinic Ward.

The KDIGO classification enables AKI to be divided into three stages, namely: stage 1 (risk), stage 2 (kidney injury), and stage 3 (kidney failure), in increasing order of severity. The persistence of serum creatinine alteration ≥0.3 mg/dL in relation to the baseline value during the 48-h period of time was characterized as AKI [16].

For data collection, a structured questionnaire prepared by the researcher and based on scientific evidence [6,17,18] was used, consisting of 16 questions, with the following variables: gender, age, ethnicity, weight, Body Mass Index, hospitalization time in the medical clinic, comorbidities, renal replacement therapy during the medical clinic stay, and laboratory variables (serum creatinine and urea) extracted from the patient’s electronic medical chart.

The Charlson Comorbidity Index (CCI) was calculated for prognostic evaluation and the final score considered the sum of the weights from 0 to 6 attributed to 17 clinical conditions, with six indicating greater severity and zero, less severity. Consequently, the patients were stratified as follows: one (1), ill; two (2), moderately-ill; from three (3) to five (5), severely-ill and six (6), nearing death [19].

The patients’ baseline serum creatinine (sCr) was extracted from their medical records, according to the criterion of results released by the laboratory: creatinine from 7 to 365 days before hospital admission; in its absence, the lowest sCr value of the first 7 hospitalization days was adopted, or sCr at hospital admission or, in the absence of the others, the lowest sCr value of the first 7 hospitalization days in the medical clinic was employed [10,20].

Renal function recovery was assessed and calculated by the ratio of serum creatinine (sCr) to baseline sCr at the time of renal function assessment, according to the following criteria: (1) Total renal function recovery: when creatinine returns to the sCr baseline value; (2) Partial recovery: when sCr does not return to the baseline value but stays within a margin up to 1.5 times the baseline value; (3) No recovery: sCr stays at a value above 1.5 times in relation to the baseline [10].

The serum creatinine and urea reference values were based on the protocol of the Health Secretariat of *Distrito Federal* (Brazil): serum sCr = 0.70–1.20 mg/dL and serum urea = 10.0–50.0 mg/dL.

Data Collection Protocol

Phase I: Weekly evaluation of the clinical and laboratory records in the patient’s electronic medical chart. Selection and inclusion of the patients took place when a sustained serum creatinine increase was identified in the minimum 48-h period of time, according to the KDIGO guidelines for AKI [16,21].

Phase II: After AKI was identified, the procedures for the collection of blood samples were carried out by the professionals of the medical clinic, according to the protocol, that is, blood collection took place on a daily basis, according to the patient’s clinical stability.

Phase III: The laboratory parameters (renal function biomarkers: serum urea and creatinine) were monitored daily, for fifteen (15) days and in months 1, 2, 3 and 6 after identifying HA-AKI by consulting the electronic medical chart regardless of whether the patient was hospitalized or not.

Phase IV: At hospital discharge, a system was created for the researcher to visit the patient face-to-face to request the laboratory tests, as well as to offer the guidelines about the need to maintain laboratory collection of serum creatinine that took place at the public basic care unit nearest to the patient’s home, which allowed evaluating sustained renal function.

Phase V: After hospital discharge, during six months of follow-up, the researcher established monthly telemonitoring through phone contacts to reinforce the guidelines about collecting serum creatinine at the basic care unit. Therefore, after the laboratory results’ control and monitoring, in the presence of any changes in the results, through phone contact, the researcher referred the patient to the basic care unit nearest to their home for a medical or nursing appointment.

The primary outcome was renal function recovery and the secondary outcome was the patients’ mortality, investigated from the records in their medical charts.

For data analysis, a descriptive analysis was performed through the calculation of the absolute and relative frequencies of the quantitative and qualitative variables, central tendency (mean and median) and dispersion (standard deviation) measures. In addition, the bootstrap confidence interval was used to verify whether or not there was a significant difference between the means of numerical variables measured over time. For the multivariate analysis, the Backward method through logistic regression was adopted for the selection of the variables and a 5% significance level was considered.

In the statistical analysis, hypothesis, Chi-Square and Fisher’s Exact tests were performed to compare the short- and long-term renal function recovery and deaths. It is worth mentioning that the tests mentioned above are non-parametric hypothesis tests and, therefore, do not assume the normality of the data.

The evaluation of the missing data to identify possible biases was performed by means of the Mann–Whitney, Chi-Square and Fisher’s Exact tests.

This study was approved by the Research Ethics Committee of the Health Sciences Teaching and Research Foundation/State Health Secretariat, CAAE: 51576215.8.0000.5553, according to Resolution 466/2012. The study participants signed the Free and Informed Consent Form (FICF).

## 3. Results

Of the 1546 patients, 202 (13.06%) were identified as with Acute Kidney Injury after being hospitalized in the Medical Clinic. Most of them were male (56.44%), aged individuals 65.5 (52.00–77.00) years old, and their Body Mass Index (BMI) was 25.08 (21.47–30.06) kg/m^2^.

The most frequent comorbidities were Systemic Arterial Hypertension (74.26%), Diabetes Mellitus (50%), heart diseases (48.02%) and respiratory diseases (34.65%). Hospitalization time in the Medical Clinic was long (39.57 ± 74.47 days).

The predominant clinical outcome was hospital discharge (62.87%), even if 27.23% of the patients evolved to death during the hospitalization and 26.24% presented this outcome after hospital discharge, which totaled 53.47% of deaths in the six-month period (Table 1).

The patients’ severity profile according to Charlson Comorbidity Index (CCI) varied predominantly from 2 to 6, which revealed moderate to serious severity (87.13%), and 31.68% were classified in the most severe stages (CCI > 4). However, when assessing AKI severity, the pattern that was noticed varied from mild (KDIGO 1) to moderate (KDIGO 2) in 70.29% of the patients. KDIGO 3 (kidney failure) was identified in 29.70% of the patients.

**Figure 1:**
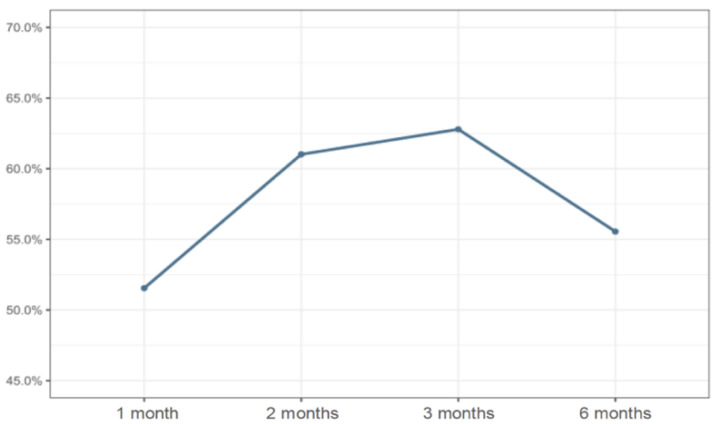
Percentage of patients with hospital-acquired Acute Kidney Injury that recovered their renal function as time progressed. Brasília (Brazil), 2017–2019.

The incidence of renal function recovery varied throughout the six months of follow-up and reached progressively high levels in the second and the third months: between 61.02% and 62.79%. However, as time progressed, specifically in the sixth month, there was a reduction in the patients’ renal recovery percentage (56%) (Figure 1).

In the detailed evaluation, it was noticed that, in the first and sixth months, the rates of renal function non-recovery were more expressive (48.45% vs. 44.44%) when compared to month 2 (38.98%) and month 3 (37.21%). In percentage terms, full renal function recovery was proportional across the different months, varying from 22.22% to 28.81% (Table 2).

**Figure 2:**
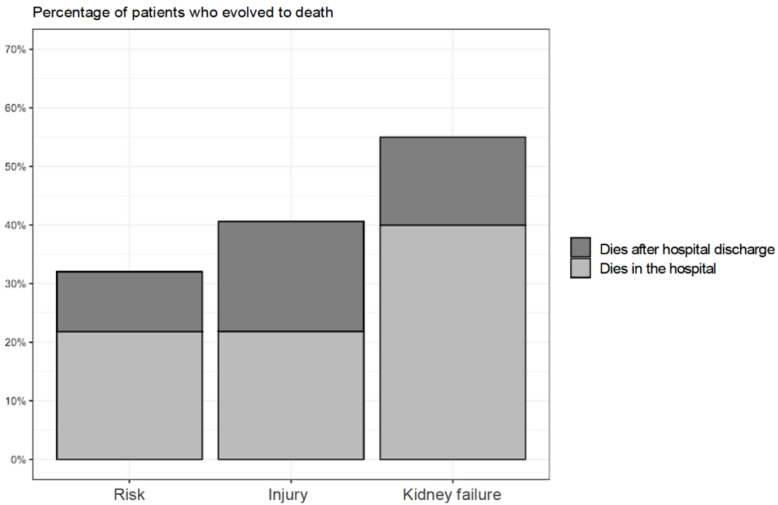
Percentage of patients who evolved to death according to Acute Kidney Injury severity during hospitalization and after discharge from the Medical Clinic (n = 202). Brasília (Brazil), 2017–2019.

It was possible to identify that the death of the patients at risk for AKI (KDIGO 1) in the in-hospital and post-discharge periods (21.79% vs. 10.26%) was lower when compared to KDIGO 3 (kidney failure) patients in the same periods (40.00% vs.15.00%) (Figure 2).

Until the third month of follow-up, the risk stage (KDIGO 1) in relation to the others (KDIGO 2 and 3) predominated with percentages from 70.00% to 73.68%. Renal recovery was significant for KDIGO 1 patients in the first month of follow-up (*p* = 0.002), unlike in the other months.

When present, renal function recovery in the first month of follow-up was a protection factor against in-hospital death (OR 0.24; 95% CI 0.09–0.61; *p*-value = 0.038) and against death in the six-month follow period (OR 0.24; 95% CI 0.09–0.61; *p*-value = 0.003) (Table 3).

## 4. Discussion

This cohort evaluated different renal function recovery patterns and their impact on the mortality of non-critical patients with hospital-acquired Acute Kidney Injury in the short- and long-term. The results showed that predominantly elderly, male and black-skinned patients with a higher severity according to the Charlson Comorbidity Index rates evolved with a tendency to early renal recovery, that is, from the first to the third month of hospitalization at the medical clinic. It is added that the patients who recovered their renal function at the first hospitalization month evolved with fewer chances of dying (*p* = 0.038).

Renal recovery has been presented as a protective factor in relation to mortality when the patient presents HA-AKI since, when there is renal function recovery, it is possible to observe better outcomes [22,23], as verified in this study. A cohort research study carried out in the United States with 16,968 patients showed that the recovery of renal impairment is related to survival higher than 90% in one year, while the group with non-recovered renal impairment had survival lower than 40% [4].

The consequences of renal recovery affect the mortality rates that tend to increase when there is a progression of the initial stages of renal dysfunction and, consequently, longer hospitalization time and worse prognosis. However, when there is a reversal in renal impairment in the short-term, that is, up to 72 h after its onset, it is still possible to observe better outcomes in the long-term [24,25]. Therefore, renal function recovery 48 h after the initial event has been shown as a forecast of quick AKI reversal and, consequently, prognostic improvement [10]. In this study, when recovery took place in the first hospitalization month, a promising prognosis was verified (*p* = 0.038) in relation to the patients who evolved with renal recovery after hospital discharge (*p* = 0.083).

It was verified that the patients of this study were older (64.18 years old) on average and had multimorbidities, such as arterial hypertension (SAH) (74.26%) and diabetes mellitus (DM) (50.00%), chronic conditions that increase the risk for AKI. It was observed that even patients in the risk stage (KDIGO 1) evolved with expressive total mortality (32%) in relation to KDIGO 3 patients (55%). The physiological cell aging in conjunction with the chronic non-communicable diseases evidenced in our study favor changes in renal structure and function, which may culminate in AKI [25]. It is highlighted that SAH as a pathology, in the long-term damages the filtering units of the kidneys, the nephrons, a complication that limits or precludes the removal of waste and excess liquid in the blood, predisposing to AKI [25]; whereas DM triggers a progressive increase in the urinary excretion of albumin, with a subsequent decrease of renal glomerular filtration [26].

The renal function recovery pattern can undergo changes due to additional injuries and recurrent exposure to risk factors during hospitalization [27], which characterizes the significant recovery of KDIGO 1 patients in the first month of follow-up, due to the shorter exposure time and lower AKI severity, differing from the other stages (KDIGO 2 and 3) who evolved with higher mortality due to greater short- and long-term renal impairment, evidencing the need to monitor the patient even after hospital discharge. In this study, patients with greater renal impairment (KDIGO 2 and 3) and with non-recovery of renal function during the six months of follow-up presented a higher tendency for the worst clinical outcome (death). This finding was also observed in a retrospective study, which pointed out that patients who did not recover their renal function until hospital discharge were two times more likely to die up to one year after hospital discharge (59%) [4].

Mortality in HA-AKI presents a frequency associated with a prolonged hospitalization period and chronic kidney disease [25], a condition that was also evidenced in our study, where 37.21% of the patients did not present renal function recovery after three months of AKI, representing the persistence of this syndrome and justifying the hospitalization time of 22 (13.00–43.00) days. Therefore, it seems evident that AKI recovery does not occur completely in all situations and that a lot of these patients evolve to CKD or terminal kidney disease [17].

The extension of the time spent in the medical clinic ward (22 (13.00–43.00) days) in our study can be associated with the severity of the patients with AKI, as well as with the metabolic changes resulting from renal impairment. A literature review that addressed AKI in non-critical patients supports our findings; it identified that, among the patients with AKI, hospitalization time is related to in-hospital mortality in the 30-day period [28].

It cannot be underestimated that, despite the therapeutic advances in the past few decades, the overall mortality of patients with AKI is still around 50% and can reach 80%, depending on the clinical conditions, comorbidities and need for renal replacement therapy [29,30].

The predominance of CCI scores above two evidence higher clinical severity and subsequent worse outcomes in the patients, a condition that was also observed in diverse scientific evidence [31,32]. In this study, it was verified that 4.46% of the patients needed Renal Replacement Therapy (RRT) during their stay at the medical clinic and that 1.18% remained dialysis-dependent after hospital discharge. It is highlighted that 27.23% evolved to death during the in-hospital period and 14.36% after discharge, which confirms the severity evidenced by the Charlson Comorbidity Index. It is also noteworthy that, even if it is a treatment, RRT has been pointed out as an independent risk factor for mortality during hospitalization [33,34].

Most of the AKI causes are avoidable and knowing the patients’ clinical profile and the factors associated with mortality among the HA-AKI patients can subsidize early and individualized interventions, such as the correction of hypovolemia and hypotension, in addition to the discontinuation of nephrotoxic agents or the correction of hyperglycemia [25]. In addition to these interventions, evidencing indicators that facilitate the recognition of the patients’ profile in the non-critical care unit can contribute to the elaboration of clinical protocols whose goal is to avoid the onset and progression of renal impairment and, therefore, mortality.

The limitations of this single center study are related to the insufficiency of data in the patients’ electronic records, the impossibility of measuring urinary volume; the non-adherence of the participating patients to the study to evaluate their renal function after hospital discharge, and patient losses, especially in the sixth month. These conditions also limited the possibility of generalizing the results. However, it is possible to consider that the data obtained can contribute and promote gains in the care process offered by the multidisciplinary team, regarding the elaboration and implementation of individualized intervention strategies, based on the patient’s need, whose goal is to limit or preclude AKI progression and mortality, favoring the reduction of the burden and economic load of this syndrome in the public health system, considering that there is still no specific and consensual treatment for Acute Kidney Injury at the global level.

## 5. Conclusions

The incidence of renal function recovery ranged throughout the six months of follow-up and reached progressively high levels in the second and third months of monitoring. In the detailed evaluation, the persistence of renal impairment and, consequently, of a condition of non-recovered renal function in the short- and long-term showed that, in the first month, and sixth months of follow up, recovery was higher than in the second and third months.

It was possible to verify that death seems to be higher in HA-AKI patients with higher severity, as it was less frequent in patients at risk for AKI (KDIGO 1) during the in-hospital and after-discharge periods when compared to KDIGO 3 patients, that is, with kidney failure in the same periods.

## Figures and Tables

**Table 1:** Clinical and demographic characteristics of the patients with hospital-acquired Acute Kidney Injury (n = 202). Brasília (Brazil), 2017–2019.

Variables	HA-AKI
**Gender** (n%)	
Male	114 (56.44)
Female	88 (43.56)
Age (years old) Median (25–75)	65.5 (52.00–77.00)
BMI (kg/m^2^) Median (25–75)	25.08 (21.47–30.06)
Blood transfusion n(%)	26 (12.94)
Hospitalization time in the Medical Clinic (days) Median (25–75)	22 (13.00–43.00)
**Ethnicity** n(%)	
White	39 (19.31)
Black	156 (77.23)
Indigenous	7 (3.47)
**Marital status** n(%)	
Single	51 (25.63)
Married	94 (47.24)
Widowed	43 (21.61)
Divorced	11 (5.53)
**Level of consciousness** n(%)	
Conscious	150 (74.26)
Torporous	18 (8.91)
Comatose	16 (7.92)
Confused	18 (8.91)
**Comorbidities** n(%)	
Systemic Arterial Hypertension	150 (74.26)
Diabetes Mellitus	101 (50.00)
Heart diseases	97 (48.02)
Respiratory diseases	70 (34.65)
Others	54 (26.74)
**Oxygen therapy** n(%)	
Room air	117 (57.92)
Nasal cannula	30 (14.85)
O_2_ mask	18 (8.91)
Non-invasive ventilation	11 (5.45)
TCT (macronebulization)	26 (12.87)
**Outcome** n(%)	
Renal Replacement Therapy (RRT) while in the MC	9 (4.46)
Dies in the hospital	55 (27.23)
High	127 (62.87)
Is transferred to another hospitalization unit	19 (9.40)
Stays hospitalized in the Medical Clinic	1 (0.50)
Dies after hospital discharge	29 (14.36)
Depends on RRT after discharge from the Medical Clinic	2 (1.18)

**Table 2:** Short- and long-term (1, 2, 3 and 6 months) full and/or partial renal function recovery in patients with hospital-acquired Acute Kidney Injury. Brasília (Brazil), 2017–2019.

Recovery/Time	n (%) *
**Month 1 (n = 97)**	
No recovery	47 (48.45)
Partial recovery	28 (28.87)
Full recovery	22 (22.68)
**Month 2 (n = 59)**	
No recovery	23 (38.98)
Partial recovery	19 (32.20)
Full recovery	17 (28.81)
**Month 3 (n = 43)**	
No recovery	16 (37.21)
Partial recovery	17 (39.53)
Full recovery	10 (23.26)
**Month 6 (n = 27)**	
No recovery	12 (44.44)
Partial recovery	9 (33.33)
Full recovery	6 (22.22)

* Percentage of the total number of patients included per month. Losses occurred during follow-up due to deaths and no serum creatinine data available.

**Table 3:** Association between short- and long-term renal function recovery and deaths among the patients with hospital-acquired Acute Kidney Injury (n = 202). Brasília (Brazil), 2017–2019.

Variables	Deaths in the Hospital			Deaths after Hospital Discharge			Deaths in the 6 Month Period		
	OR	95% CI	*p*-Value	OR	95% CI	*p*-Value	OR	95% CI	*p*-Value
Recovery at Month 1	0.24	[0.06; 0.92]	0.038	0.36	[0.12; 1.14]	0.083	0.24	[0.09; 0.61]	0.003
Recovery at Month 2	0.28	[0.05; 1.67]	0.162	1.69	[0.30; 9.56]	0.551	0.68	[0.20; 2.37]	0.549
Recovery at Month 3	0.12	[0.10; 14.39]	0.886	0.18	[0.03; 1.05]	0.057	0.29	[0.07; 1.26]	0.098
Recovery at Month 6	-	-	0.997	-	-	0.997	0.79	[0.04; 14.03]	0.870

## Data Availability

Not applicable.
